# 2-Amino-4-(4-chloro­phen­yl)-4*H*-chromeno[8,7-*b*]pyridine-3-carbonitrile

**DOI:** 10.1107/S1600536813005217

**Published:** 2013-03-02

**Authors:** Abd El-Galil E. Amr, Ahmed M. El-Agrody, Nermien M. Sabry, Seik Weng Ng, Edward R. T. Tiekink

**Affiliations:** a Drug Exploration & Development Chair (DEDC), College of Pharmacy, King Saud University, Riyadh 11451, Saudi Arabia; b Applied Organic Chemistry Department, National Research Center, Dokki 12622, Cairo, Egypt; c Chemistry Department, Faculty of Science, King Khalid University, Abha 61413, PO Box 9004, Saudi Arabia; d Department of Chemistry, University of Malaya, 50603 Kuala Lumpur, Malaysia; e Chemistry Department, Faculty of Science, King Abdulaziz University, PO Box 80203 Jeddah, Saudi Arabia

## Abstract

The asymmetric unit of the title compound, C_19_H_12_ClN_3_O, contains two mol­ecules with similar conformations. The 14 non-H atoms comprising the 4*H*-chromeno[8,7-*b*]pyridine residue are essentially coplanar (r.m.s. deviations = 0.037 and 0.042 Å for the two mol­ecules) and the main difference between them is seen in the twist about the bond linking the main residue to the attached chloro­benzene rings [dihedral angles = 79.01 (12) and 76.22 (11)° for the two mol­ecules]. Zigzag supra­molecular chains along the *a*-axis direction mediated by amino–pyridine N—H⋯N hydrogen bonds feature in the crystal packing; these are connected into a three-dimensional architecture by C—H⋯π inter­actions and Cl⋯Cl contacts [Cl⋯Cl = 3.3896 (14) Å].

## Related literature

For background to the chemistry and biological activity of 4*H*-pyran derivatives, see: Al-Ghamdi *et al.* (2012 ▶); El-Agrody *et al.* (2012 ▶). For the structure of the 2-chloro analogue, see: Wang *et al.* (2003 ▶).
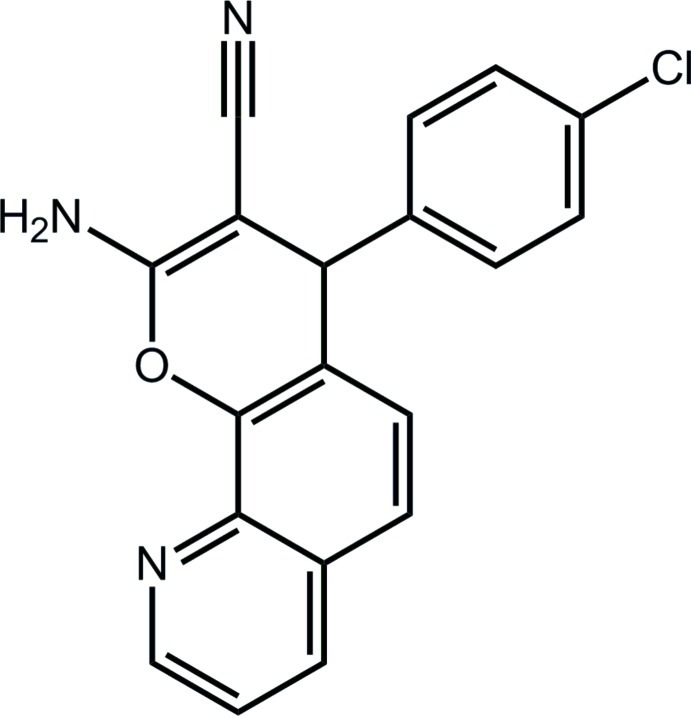


## Experimental

### 

#### Crystal data

C_19_H_12_ClN_3_O
*M* *_r_* = 333.77
Monoclinic, 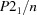

*a* = 6.5311 (8) Å
*b* = 35.129 (3) Å
*c* = 14.0903 (14) Å
β = 101.740 (11)°
*V* = 3165.2 (6) Å^3^
*Z* = 8
Mo *K*α radiation
μ = 0.25 mm^−1^
*T* = 295 K
0.30 × 0.20 × 0.05 mm

#### Data collection

Agilent SuperNova Dual diffractometer with an Atlas detector
Absorption correction: multi-scan (*CrysAlis PRO*; Agilent, 2011 ▶) *T*_min_ = 0.833, *T*_max_ = 1.000
20646 measured reflections
7326 independent reflections
3471 reflections with *I* > 2σ(*I*)
*R*_int_ = 0.068

#### Refinement

*R*[*F* ^2^ > 2σ(*F* ^2^)] = 0.061
*wR*(*F* ^2^) = 0.164
*S* = 1.01
7326 reflections
450 parameters
H atoms treated by a mixture of independent and constrained refinement
Δρ_max_ = 0.21 e Å^−3^
Δρ_min_ = −0.32 e Å^−3^

### 

Data collection: *CrysAlis PRO* (Agilent, 2011 ▶); cell refinement: *CrysAlis PRO*; data reduction: *CrysAlis PRO*; program(s) used to solve structure: *SHELXS97* (Sheldrick, 2008 ▶); program(s) used to refine structure: *SHELXL97* (Sheldrick, 2008 ▶); molecular graphics: *ORTEP-3 for Windows* (Farrugia, 2012 ▶), *QMol* (Gans & Shalloway, 2001 ▶) and *DIAMOND* (Brandenburg, 2006 ▶); software used to prepare material for publication: *publCIF* (Westrip, 2010 ▶).

## Supplementary Material

Crystal structure: contains datablock(s) global, I. DOI: 10.1107/S1600536813005217/hb7046sup1.cif

Structure factors: contains datablock(s) I. DOI: 10.1107/S1600536813005217/hb7046Isup2.hkl

Supplementary material file. DOI: 10.1107/S1600536813005217/hb7046Isup3.cml

Additional supplementary materials:  crystallographic information; 3D view; checkCIF report

## Figures and Tables

**Table 1:** Hydrogen-bond geometry (Å, °) *Cg*1 and *Cg*2 are the centroids of the N4,C20–C23,C28 and C33–C38 rings, respectively.

*D*—H⋯*A*	*D*—H	H⋯*A*	*D*⋯*A*	*D*—H⋯*A*
N2—H1⋯N4^i^	0.84 (3)	2.34 (4)	3.172 (4)	174 (3)
N2—H2⋯N5^i^	0.89 (3)	2.61 (3)	3.308 (5)	136 (2)
N5—H3⋯N1	0.87 (3)	2.15 (3)	3.014 (3)	173 (3)
C18—H18⋯*Cg*1^ii^	0.93	2.80	3.650 (3)	152
C24—H24⋯*Cg*2^iii^	0.93	2.74	3.668 (3)	174

Symmetry codes: (i) 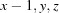; (ii) 
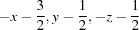
; (iii) 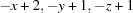.

**Fig. 1.**
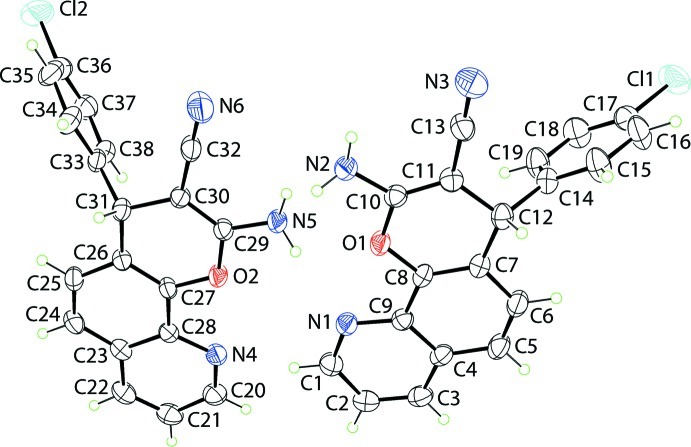
The molecular structures of the two independent molecules comprising the asymmetric unit in (I) showing displacement ellipsoids at the 35% probability level.

**Fig. 2.**
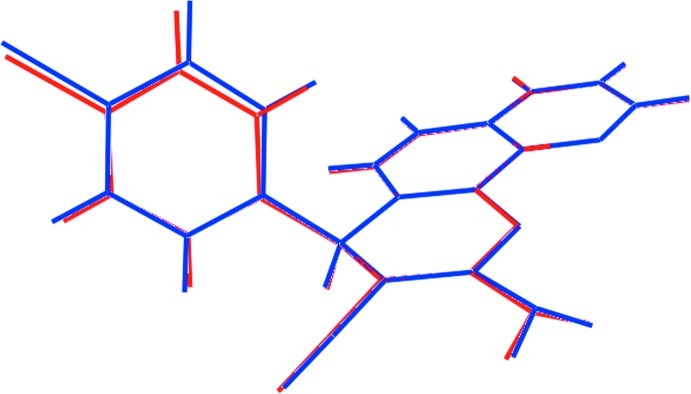
Overlay diagram of the two independent molecules in (I) with the inverted N1-containing molecule illustrated in red. The molecules are overlaid so that the pyridyl rings are superimposed.

**Fig. 3.**
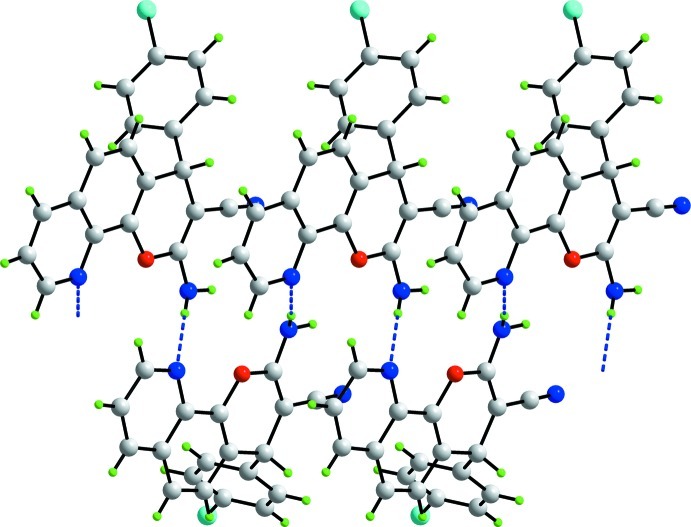
A view of the supramolecular chain along the a axis in (I) sustained by N—H···N hydrogen bonding, shown as blue dashed lines.

**Fig. 4.**
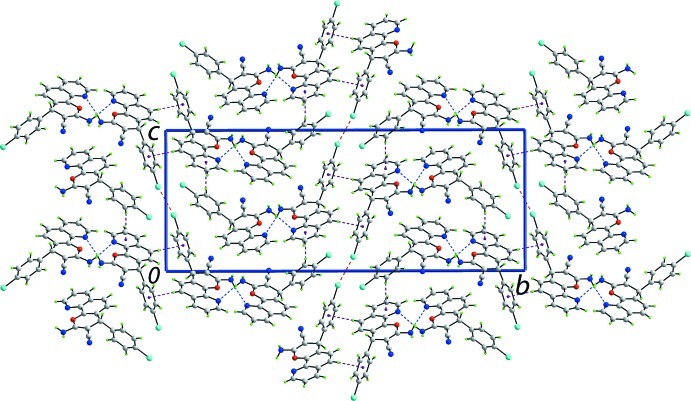
A view in projection down the a axis of the crystal packing in (I). The N—H···N, C—H···π and Cl···Cl interactions are shown as blue, purple and orange dashed lines, respectively.

## supplementary crystallographic information

### Comment

Motivated by their biological activities and in continuation of an on-going programme on the chemistry of 4*H*-pyran derivatives (Al-Ghamdi *et al.*, 2012; El-Agrody *et al.*, 2012), the synthesis and crystal structure determination of (I) is reported.

Two independent molecules comprise the asymmetric unit of (I), Fig. 1. As illustrated in Fig. 2, where the O2-containing molecule is super-imposed upon the inverse of the O1-containing molecule, the molecules are virtually super-imposable with differences apparent in the relationship between the 4*H*-chromeno[8,7-*b*]pyridine residue and the attached benzene ring. For the O1-containing molecule, the r.m.s. deviation of the 14 non-hydrogen atoms comprising the fused ring system is 0.037 Å, the dihedral angle between this and the benzene ring is 79.01 (12)° and the twist between these groups is manifested in the C7—C12—C14—C15 torsion angle of -132.9 (3)°. The comparable values for the second molecule are 0.042 Å, 76.22 (11)° and 149.0 (3)°, respectively. The observed conformation is in accord with that established previously for the 2-chloro analogue (Wang *et al.*, 2003).

The most prominent feature of the crystal packing is the formation of supramolecular zigzag chains along the *a* axis mediated by (amino)N—H···N(pyridyl) hydrogen bonding, Fig. 3 and Table 1. Whereas the second N1-bound H2 atom forms a weak interaction to the N5 atom, Table 1, reinforcing the chain, the second N2-bound H4 atom does not form a significant intermolecular interaction. The chains are connected into a three-dimensional architecture by C—H···π interactions along with Cl2···Cl2^i^ contacts [Cl2···Cl2^i^ = 3.3896 (14) Å for *i*: 2 - *x*, 1 - *y*, 2 - *z*], Fig. 4.

### Experimental

A solution of 8-hydroxyquinoline (0.01 mol) in EtOH (30 ml) was treated with α-cyano-*p*-chlorocinnamonitrile (0.01 mol) and piperidine (0.5 ml). The reaction mixture was heated for 60 minutes by which time complete precipitation occurred. The solid product was collected by filtration and recrystallized from ethanol to give yellow prisms of the title compound, (I); *M*.pt: 522–523 K.

### Refinement

The C-bound H atoms were geometrically placed (C–H = 0.93–0.98 Å) and refined as riding with *U_iso_*(H) = 1.2*U_eq_*(C). The N-bound-H atoms were refined freely.

### Crystal data

**Table:**

C_19_H_12_ClN_3_O	*F*(000) = 1376
*M**_r_* = 333.77	*D*_x_ = 1.401 Mg m^−^^3^
Monoclinic, *P*2_1_/*n*	Mo *K*α radiation, λ = 0.71073 Å
Hall symbol: -P 2yn	Cell parameters from 2452 reflections
*a* = 6.5311 (8) Å	θ = 2.9–27.5°
*b* = 35.129 (3) Å	µ = 0.25 mm^−^^1^
*c* = 14.0903 (14) Å	*T* = 295 K
β = 101.740 (11)°	Prism, yellow
*V* = 3165.2 (6) Å^3^	0.30 × 0.20 × 0.05 mm
*Z* = 8	

### Data collection

**Table:**

Agilent SuperNova Dual diffractometer with an Atlas detector	7326 independent reflections
Radiation source: SuperNova (Mo) X-ray Source	3471 reflections with *I* > 2σ(*I*)
Mirror monochromator	*R*_int_ = 0.068
Detector resolution: 10.4041 pixels mm^-1^	θ_max_ = 27.6°, θ_min_ = 3.0°
ω scan	*h* = −8→8
Absorption correction: multi-scan (*CrysAlis PRO*; Agilent, 2011)	*k* = −44→45
*T*_min_ = 0.833, *T*_max_ = 1.000	*l* = −18→18
20646 measured reflections	

### Refinement

**Table:**

Refinement on *F*^2^	Secondary atom site location: difference Fourier map
Least-squares matrix: full	Hydrogen site location: inferred from neighbouring sites
*R*[*F*^2^ > 2σ(*F*^2^)] = 0.061	H atoms treated by a mixture of independent and constrained refinement
*wR*(*F*^2^) = 0.164	*w* = 1/[σ^2^(*F*_o_^2^) + (0.0527*P*)^2^] where *P* = (*F*_o_^2^ + 2*F*_c_^2^)/3
*S* = 1.01	(Δ/σ)_max_ = 0.001
7326 reflections	Δρ_max_ = 0.21 e Å^−^^3^
450 parameters	Δρ_min_ = −0.32 e Å^−^^3^
0 restraints	Extinction correction: *SHELXL97* (Sheldrick, 2008), Fc^*^=kFc[1+0.001xFc^2^λ^3^/sin(2θ)]^-1/4^
Primary atom site location: structure-invariant direct methods	Extinction coefficient: 0.0023 (4)

### Special details

**Table:**

Geometry. All e.s.d.'s (except the e.s.d. in the dihedral angle between two l.s. planes) are estimated using the full covariance matrix. The cell e.s.d.'s are taken into account individually in the estimation of e.s.d.'s in distances, angles and torsion angles; correlations between e.s.d.'s in cell parameters are only used when they are defined by crystal symmetry. An approximate (isotropic) treatment of cell e.s.d.'s is used for estimating e.s.d.'s involving l.s. planes.
Refinement. Refinement of *F*^2^ against ALL reflections. The weighted *R*-factor *wR* and goodness of fit *S* are based on *F*^2^, conventional *R*-factors *R* are based on *F*, with *F* set to zero for negative *F*^2^. The threshold expression of *F*^2^ > σ(*F*^2^) is used only for calculating *R*-factors(gt) *etc*. and is not relevant to the choice of reflections for refinement. *R*-factors based on *F*^2^ are statistically about twice as large as those based on *F*, and *R*- factors based on ALL data will be even larger.

### Fractional atomic coordinates and isotropic or equivalent isotropic displacement parameters (Å^2^)

**Table:**

	*x*	*y*	*z*	*U*_iso_*/*U*_eq_	
Cl1	0.14716 (19)	0.04691 (3)	0.61417 (7)	0.0887 (4)	
Cl2	0.8629 (2)	0.47842 (3)	0.89931 (6)	0.1067 (5)	
O1	0.1506 (3)	0.25977 (5)	0.34064 (13)	0.0470 (5)	
O2	0.6996 (3)	0.35767 (5)	0.38366 (13)	0.0464 (5)	
N1	0.4684 (4)	0.27492 (6)	0.25590 (15)	0.0455 (6)	
N2	−0.0484 (5)	0.28690 (7)	0.43039 (19)	0.0494 (7)	
N3	−0.3636 (5)	0.21391 (8)	0.5043 (2)	0.0800 (10)	
N4	1.0071 (4)	0.35491 (6)	0.28969 (16)	0.0475 (6)	
N5	0.4811 (5)	0.31956 (7)	0.4397 (2)	0.0500 (7)	
N6	0.2277 (5)	0.37488 (8)	0.5911 (2)	0.0873 (11)	
C1	0.6186 (5)	0.28248 (8)	0.2095 (2)	0.0512 (8)	
H1A	0.6696	0.3073	0.2126	0.061*	
C2	0.7079 (5)	0.25603 (9)	0.15559 (19)	0.0528 (8)	
H2A	0.8134	0.2632	0.1237	0.063*	
C3	0.6363 (5)	0.21963 (9)	0.15116 (19)	0.0511 (8)	
H3A	0.6953	0.2014	0.1171	0.061*	
C4	0.4729 (5)	0.20941 (8)	0.19792 (18)	0.0445 (7)	
C5	0.3886 (5)	0.17228 (8)	0.1979 (2)	0.0549 (9)	
H5	0.4396	0.1529	0.1641	0.066*	
C6	0.2346 (5)	0.16457 (8)	0.2465 (2)	0.0542 (8)	
H6	0.1832	0.1399	0.2458	0.065*	
C7	0.1495 (5)	0.19300 (7)	0.29835 (18)	0.0421 (7)	
C8	0.2278 (5)	0.22901 (7)	0.29687 (18)	0.0403 (7)	
C9	0.3921 (5)	0.23850 (7)	0.24918 (17)	0.0407 (7)	
C10	−0.0007 (5)	0.25317 (8)	0.39165 (18)	0.0409 (7)	
C11	−0.0810 (5)	0.21862 (8)	0.40245 (19)	0.0437 (7)	
C12	−0.0248 (5)	0.18351 (7)	0.35028 (19)	0.0458 (7)	
H12	−0.1477	0.1772	0.3003	0.055*	
C13	−0.2384 (6)	0.21591 (8)	0.4580 (2)	0.0531 (8)	
C14	0.0212 (5)	0.14887 (8)	0.4167 (2)	0.0453 (7)	
C15	−0.1253 (6)	0.12104 (9)	0.4144 (2)	0.0702 (10)	
H15	−0.2530	0.1233	0.3712	0.084*	
C16	−0.0887 (7)	0.08930 (9)	0.4751 (3)	0.0751 (11)	
H16	−0.1905	0.0706	0.4728	0.090*	
C17	0.0984 (7)	0.08624 (9)	0.5375 (2)	0.0579 (9)	
C18	0.2466 (6)	0.11359 (10)	0.5426 (2)	0.0690 (10)	
H18	0.3736	0.1113	0.5863	0.083*	
C19	0.2072 (6)	0.14497 (9)	0.4821 (2)	0.0637 (9)	
H19	0.3086	0.1638	0.4858	0.076*	
C20	1.1555 (5)	0.35407 (9)	0.2385 (2)	0.0552 (9)	
H20	1.1859	0.3308	0.2131	0.066*	
C21	1.2701 (5)	0.38601 (10)	0.2201 (2)	0.0584 (9)	
H21	1.3715	0.3838	0.1827	0.070*	
C22	1.2309 (5)	0.42014 (9)	0.2577 (2)	0.0562 (9)	
H22	1.3058	0.4416	0.2461	0.067*	
C23	1.0766 (5)	0.42312 (8)	0.31426 (19)	0.0464 (8)	
C24	1.0277 (5)	0.45694 (8)	0.3591 (2)	0.0540 (9)	
H24	1.0983	0.4793	0.3510	0.065*	
C25	0.8789 (5)	0.45719 (7)	0.4137 (2)	0.0482 (8)	
H25	0.8528	0.4797	0.4439	0.058*	
C26	0.7618 (5)	0.42418 (7)	0.42611 (17)	0.0394 (7)	
C27	0.8066 (5)	0.39158 (7)	0.38100 (18)	0.0381 (7)	
C28	0.9661 (5)	0.38958 (7)	0.32597 (17)	0.0400 (7)	
C29	0.5538 (5)	0.35562 (7)	0.43977 (18)	0.0404 (7)	
C30	0.5022 (5)	0.38585 (7)	0.48884 (18)	0.0401 (7)	
C31	0.5956 (5)	0.42540 (7)	0.48574 (18)	0.0405 (7)	
H31	0.4841	0.4422	0.4525	0.049*	
C32	0.3499 (6)	0.37995 (8)	0.5446 (2)	0.0537 (8)	
C33	0.6695 (5)	0.44071 (7)	0.5878 (2)	0.0434 (7)	
C34	0.5327 (6)	0.46012 (8)	0.6330 (2)	0.0602 (9)	
H34	0.3988	0.4657	0.5987	0.072*	
C35	0.5929 (7)	0.47155 (9)	0.7299 (3)	0.0708 (11)	
H35	0.4991	0.4845	0.7599	0.085*	
C36	0.7888 (7)	0.46368 (9)	0.7799 (2)	0.0644 (10)	
C37	0.9283 (6)	0.44463 (8)	0.7368 (2)	0.0613 (9)	
H37	1.0626	0.4395	0.7713	0.074*	
C38	0.8671 (5)	0.43299 (8)	0.6410 (2)	0.0521 (8)	
H38	0.9613	0.4197	0.6120	0.063*	
H1	−0.032 (6)	0.3060 (10)	0.397 (2)	0.081 (12)*	
H2	−0.157 (5)	0.2859 (8)	0.460 (2)	0.064 (11)*	
H3	0.469 (5)	0.3081 (9)	0.384 (2)	0.073 (11)*	
H4	0.369 (5)	0.3152 (8)	0.467 (2)	0.061 (10)*	

### Atomic displacement parameters (Å^2^)

**Table:**

	*U*^11^	*U*^22^	*U*^33^	*U*^12^	*U*^13^	*U*^23^
Cl1	0.1187 (10)	0.0705 (6)	0.0826 (6)	0.0147 (6)	0.0336 (6)	0.0305 (5)
Cl2	0.1573 (13)	0.1087 (8)	0.0572 (5)	−0.0307 (8)	0.0293 (6)	−0.0242 (5)
O1	0.0519 (15)	0.0330 (10)	0.0612 (11)	−0.0010 (10)	0.0238 (11)	−0.0079 (8)
O2	0.0496 (14)	0.0337 (10)	0.0638 (12)	−0.0065 (10)	0.0302 (11)	−0.0081 (9)
N1	0.0483 (18)	0.0410 (13)	0.0499 (13)	−0.0018 (12)	0.0161 (13)	−0.0042 (11)
N2	0.059 (2)	0.0390 (14)	0.0557 (15)	0.0046 (14)	0.0237 (15)	−0.0015 (12)
N3	0.074 (2)	0.085 (2)	0.090 (2)	−0.0125 (19)	0.038 (2)	−0.0017 (17)
N4	0.0521 (18)	0.0416 (13)	0.0539 (14)	0.0040 (13)	0.0228 (13)	0.0030 (11)
N5	0.058 (2)	0.0373 (13)	0.0627 (16)	−0.0117 (13)	0.0308 (16)	−0.0083 (12)
N6	0.093 (3)	0.0567 (18)	0.135 (3)	0.0027 (18)	0.077 (2)	0.0025 (18)
C1	0.053 (2)	0.0495 (17)	0.0538 (17)	−0.0009 (16)	0.0169 (17)	0.0005 (14)
C2	0.052 (2)	0.064 (2)	0.0446 (15)	0.0023 (18)	0.0150 (15)	0.0032 (15)
C3	0.057 (2)	0.0555 (19)	0.0419 (15)	0.0101 (17)	0.0140 (16)	−0.0029 (14)
C4	0.050 (2)	0.0438 (16)	0.0388 (14)	0.0091 (15)	0.0070 (14)	−0.0051 (12)
C5	0.064 (3)	0.0407 (16)	0.0620 (18)	0.0061 (17)	0.0180 (18)	−0.0129 (14)
C6	0.062 (2)	0.0406 (16)	0.0614 (18)	−0.0049 (17)	0.0148 (18)	−0.0123 (14)
C7	0.044 (2)	0.0355 (14)	0.0461 (15)	0.0006 (14)	0.0074 (14)	−0.0048 (12)
C8	0.043 (2)	0.0335 (14)	0.0431 (14)	0.0026 (14)	0.0069 (14)	−0.0067 (12)
C9	0.041 (2)	0.0410 (15)	0.0395 (14)	0.0021 (14)	0.0073 (14)	−0.0007 (12)
C10	0.041 (2)	0.0410 (15)	0.0413 (14)	0.0035 (15)	0.0103 (14)	−0.0009 (12)
C11	0.038 (2)	0.0440 (16)	0.0491 (16)	0.0006 (15)	0.0102 (15)	0.0001 (13)
C12	0.042 (2)	0.0415 (15)	0.0502 (16)	−0.0049 (15)	0.0015 (15)	−0.0017 (13)
C13	0.052 (2)	0.0469 (18)	0.0615 (19)	−0.0057 (17)	0.0152 (18)	0.0030 (14)
C14	0.043 (2)	0.0384 (15)	0.0537 (17)	−0.0019 (15)	0.0079 (16)	−0.0024 (13)
C15	0.061 (3)	0.057 (2)	0.085 (2)	−0.016 (2)	−0.004 (2)	0.0148 (18)
C16	0.079 (3)	0.054 (2)	0.089 (2)	−0.025 (2)	0.009 (2)	0.0132 (19)
C17	0.074 (3)	0.0490 (18)	0.0531 (18)	0.004 (2)	0.0177 (19)	0.0036 (15)
C18	0.065 (3)	0.068 (2)	0.067 (2)	−0.003 (2)	−0.0054 (19)	0.0121 (18)
C19	0.055 (2)	0.0527 (19)	0.077 (2)	−0.0133 (18)	−0.0028 (19)	0.0052 (17)
C20	0.059 (2)	0.0555 (19)	0.0569 (17)	0.0111 (18)	0.0257 (18)	0.0023 (15)
C21	0.053 (2)	0.072 (2)	0.0566 (18)	0.0007 (19)	0.0259 (17)	0.0088 (17)
C22	0.052 (2)	0.060 (2)	0.0598 (18)	−0.0096 (18)	0.0187 (17)	0.0118 (16)
C23	0.049 (2)	0.0456 (16)	0.0460 (15)	−0.0031 (16)	0.0127 (15)	0.0086 (13)
C24	0.065 (3)	0.0423 (17)	0.0570 (17)	−0.0141 (17)	0.0172 (18)	0.0074 (14)
C25	0.056 (2)	0.0326 (15)	0.0572 (17)	−0.0037 (15)	0.0137 (17)	−0.0016 (13)
C26	0.0399 (19)	0.0338 (14)	0.0451 (14)	−0.0006 (14)	0.0100 (14)	0.0026 (12)
C27	0.0391 (19)	0.0304 (13)	0.0464 (15)	−0.0025 (13)	0.0122 (14)	0.0030 (11)
C28	0.044 (2)	0.0383 (14)	0.0390 (14)	0.0003 (14)	0.0103 (14)	0.0042 (12)
C29	0.041 (2)	0.0349 (14)	0.0480 (15)	−0.0028 (14)	0.0147 (14)	−0.0002 (12)
C30	0.0402 (19)	0.0363 (14)	0.0468 (15)	−0.0034 (14)	0.0158 (14)	−0.0042 (12)
C31	0.0400 (19)	0.0319 (13)	0.0505 (15)	0.0037 (14)	0.0118 (14)	−0.0024 (12)
C32	0.058 (2)	0.0343 (15)	0.076 (2)	−0.0001 (16)	0.0303 (19)	−0.0032 (14)
C33	0.049 (2)	0.0294 (13)	0.0554 (16)	0.0000 (15)	0.0186 (16)	−0.0050 (12)
C34	0.053 (2)	0.0528 (18)	0.079 (2)	−0.0001 (18)	0.0229 (19)	−0.0214 (16)
C35	0.080 (3)	0.061 (2)	0.083 (2)	−0.015 (2)	0.046 (2)	−0.0294 (19)
C36	0.084 (3)	0.058 (2)	0.0573 (19)	−0.017 (2)	0.028 (2)	−0.0092 (16)
C37	0.075 (3)	0.0530 (18)	0.0532 (18)	−0.0008 (19)	0.0072 (18)	0.0023 (15)
C38	0.059 (2)	0.0430 (16)	0.0554 (18)	0.0070 (17)	0.0150 (17)	−0.0033 (14)

### Geometric parameters (Å, º)

**Table:**

Cl1—C17	1.743 (3)	C14—C19	1.374 (4)
Cl2—C36	1.732 (3)	C15—C16	1.396 (4)
O1—C10	1.355 (3)	C15—H15	0.9300
O1—C8	1.389 (3)	C16—C17	1.357 (5)
O2—C29	1.358 (3)	C16—H16	0.9300
O2—C27	1.385 (3)	C17—C18	1.355 (4)
N1—C1	1.312 (3)	C18—C19	1.385 (4)
N1—C9	1.369 (3)	C18—H18	0.9300
N2—C10	1.367 (3)	C19—H19	0.9300
N2—H1	0.84 (3)	C20—C21	1.402 (4)
N2—H2	0.89 (3)	C20—H20	0.9300
N3—C13	1.147 (4)	C21—C22	1.356 (4)
N4—C20	1.321 (3)	C21—H21	0.9300
N4—C28	1.368 (3)	C22—C23	1.411 (4)
N5—C29	1.353 (3)	C22—H22	0.9300
N5—H3	0.87 (3)	C23—C28	1.409 (4)
N5—H4	0.91 (3)	C23—C24	1.412 (4)
N6—C32	1.145 (4)	C24—C25	1.357 (4)
C1—C2	1.400 (4)	C24—H24	0.9300
C1—H1A	0.9300	C25—C26	1.419 (4)
C2—C3	1.359 (4)	C25—H25	0.9300
C2—H2A	0.9300	C26—C27	1.370 (3)
C3—C4	1.410 (4)	C26—C31	1.502 (4)
C3—H3A	0.9300	C27—C28	1.421 (4)
C4—C9	1.414 (3)	C29—C30	1.347 (3)
C4—C5	1.416 (4)	C30—C32	1.403 (4)
C5—C6	1.354 (4)	C30—C31	1.522 (3)
C5—H5	0.9300	C31—C33	1.520 (4)
C6—C7	1.416 (4)	C31—H31	0.9800
C6—H6	0.9300	C33—C34	1.378 (4)
C7—C8	1.366 (4)	C33—C38	1.381 (4)
C7—C12	1.510 (4)	C34—C35	1.401 (5)
C8—C9	1.417 (4)	C34—H34	0.9300
C10—C11	1.343 (4)	C35—C36	1.358 (5)
C11—C13	1.417 (4)	C35—H35	0.9300
C11—C12	1.519 (4)	C36—C37	1.368 (5)
C12—C14	1.527 (4)	C37—C38	1.390 (4)
C12—H12	0.9800	C37—H37	0.9300
C14—C15	1.364 (4)	C38—H38	0.9300
			
C10—O1—C8	118.2 (2)	C17—C18—H18	120.3
C29—O2—C27	118.59 (19)	C19—C18—H18	120.3
C1—N1—C9	116.9 (2)	C14—C19—C18	121.4 (3)
C10—N2—H1	114 (2)	C14—C19—H19	119.3
C10—N2—H2	115 (2)	C18—C19—H19	119.3
H1—N2—H2	120 (3)	N4—C20—C21	124.2 (3)
C20—N4—C28	116.4 (2)	N4—C20—H20	117.9
C29—N5—H3	114 (2)	C21—C20—H20	117.9
C29—N5—H4	118.3 (19)	C22—C21—C20	119.0 (3)
H3—N5—H4	111 (3)	C22—C21—H21	120.5
N1—C1—C2	124.9 (3)	C20—C21—H21	120.5
N1—C1—H1A	117.5	C21—C22—C23	120.0 (3)
C2—C1—H1A	117.5	C21—C22—H22	120.0
C3—C2—C1	118.2 (3)	C23—C22—H22	120.0
C3—C2—H2A	120.9	C28—C23—C22	116.5 (3)
C1—C2—H2A	120.9	C28—C23—C24	118.9 (3)
C2—C3—C4	120.3 (3)	C22—C23—C24	124.6 (3)
C2—C3—H3A	119.9	C25—C24—C23	120.8 (3)
C4—C3—H3A	119.9	C25—C24—H24	119.6
C3—C4—C9	116.8 (3)	C23—C24—H24	119.6
C3—C4—C5	124.5 (3)	C24—C25—C26	122.0 (3)
C9—C4—C5	118.7 (3)	C24—C25—H25	119.0
C6—C5—C4	120.9 (3)	C26—C25—H25	119.0
C6—C5—H5	119.6	C27—C26—C25	117.0 (2)
C4—C5—H5	119.6	C27—C26—C31	122.0 (2)
C5—C6—C7	122.0 (3)	C25—C26—C31	120.9 (2)
C5—C6—H6	119.0	C26—C27—O2	123.4 (2)
C7—C6—H6	119.0	C26—C27—C28	123.0 (2)
C8—C7—C6	117.2 (3)	O2—C27—C28	113.7 (2)
C8—C7—C12	122.2 (2)	N4—C28—C23	123.8 (2)
C6—C7—C12	120.5 (2)	N4—C28—C27	118.0 (2)
C7—C8—O1	122.9 (2)	C23—C28—C27	118.2 (2)
C7—C8—C9	123.2 (2)	C30—C29—N5	127.9 (3)
O1—C8—C9	114.0 (2)	C30—C29—O2	122.4 (2)
N1—C9—C4	122.8 (3)	N5—C29—O2	109.7 (2)
N1—C9—C8	119.1 (2)	C29—C30—C32	116.8 (2)
C4—C9—C8	118.0 (2)	C29—C30—C31	124.2 (2)
C11—C10—O1	123.9 (2)	C32—C30—C31	119.0 (2)
C11—C10—N2	127.7 (3)	C26—C31—C33	114.4 (2)
O1—C10—N2	108.3 (2)	C26—C31—C30	109.2 (2)
C10—C11—C13	117.9 (3)	C33—C31—C30	110.4 (2)
C10—C11—C12	122.8 (2)	C26—C31—H31	107.5
C13—C11—C12	119.1 (3)	C33—C31—H31	107.5
C7—C12—C11	109.6 (2)	C30—C31—H31	107.5
C7—C12—C14	113.5 (2)	N6—C32—C30	178.9 (4)
C11—C12—C14	112.8 (2)	C34—C33—C38	118.0 (3)
C7—C12—H12	106.8	C34—C33—C31	120.1 (3)
C11—C12—H12	106.8	C38—C33—C31	121.7 (2)
C14—C12—H12	106.8	C33—C34—C35	120.8 (3)
N3—C13—C11	178.9 (4)	C33—C34—H34	119.6
C15—C14—C19	117.7 (3)	C35—C34—H34	119.6
C15—C14—C12	120.4 (3)	C36—C35—C34	119.7 (3)
C19—C14—C12	121.8 (3)	C36—C35—H35	120.2
C14—C15—C16	121.7 (4)	C34—C35—H35	120.2
C14—C15—H15	119.1	C35—C36—C37	120.8 (3)
C16—C15—H15	119.1	C35—C36—Cl2	119.4 (3)
C17—C16—C15	118.7 (3)	C37—C36—Cl2	119.8 (3)
C17—C16—H16	120.7	C36—C37—C38	119.3 (3)
C15—C16—H16	120.7	C36—C37—H37	120.3
C16—C17—C18	121.2 (3)	C38—C37—H37	120.3
C16—C17—Cl1	119.4 (3)	C33—C38—C37	121.4 (3)
C18—C17—Cl1	119.4 (3)	C33—C38—H38	119.3
C17—C18—C19	119.3 (3)	C37—C38—H38	119.3
			
C9—N1—C1—C2	−1.2 (4)	C28—N4—C20—C21	0.1 (5)
N1—C1—C2—C3	−0.6 (5)	N4—C20—C21—C22	1.0 (5)
C1—C2—C3—C4	1.5 (4)	C20—C21—C22—C23	−0.1 (5)
C2—C3—C4—C9	−0.7 (4)	C21—C22—C23—C28	−1.8 (4)
C2—C3—C4—C5	−179.6 (3)	C21—C22—C23—C24	178.1 (3)
C3—C4—C5—C6	178.5 (3)	C28—C23—C24—C25	0.9 (5)
C9—C4—C5—C6	−0.5 (5)	C22—C23—C24—C25	−178.9 (3)
C4—C5—C6—C7	0.7 (5)	C23—C24—C25—C26	−1.9 (5)
C5—C6—C7—C8	0.6 (5)	C24—C25—C26—C27	0.6 (4)
C5—C6—C7—C12	178.9 (3)	C24—C25—C26—C31	−179.0 (3)
C6—C7—C8—O1	176.7 (3)	C25—C26—C27—O2	−177.5 (2)
C12—C7—C8—O1	−1.6 (4)	C31—C26—C27—O2	2.2 (4)
C6—C7—C8—C9	−2.1 (4)	C25—C26—C27—C28	1.8 (4)
C12—C7—C8—C9	179.6 (3)	C31—C26—C27—C28	−178.6 (3)
C10—O1—C8—C7	3.9 (4)	C29—O2—C27—C26	−5.2 (4)
C10—O1—C8—C9	−177.2 (2)	C29—O2—C27—C28	175.5 (2)
C1—N1—C9—C4	2.1 (4)	C20—N4—C28—C23	−2.2 (4)
C1—N1—C9—C8	−179.1 (3)	C20—N4—C28—C27	179.7 (3)
C3—C4—C9—N1	−1.2 (4)	C22—C23—C28—N4	3.0 (4)
C5—C4—C9—N1	177.8 (3)	C24—C23—C28—N4	−176.8 (3)
C3—C4—C9—C8	180.0 (3)	C22—C23—C28—C27	−178.8 (3)
C5—C4—C9—C8	−1.0 (4)	C24—C23—C28—C27	1.3 (4)
C7—C8—C9—N1	−176.5 (3)	C26—C27—C28—N4	175.5 (3)
O1—C8—C9—N1	4.6 (4)	O2—C27—C28—N4	−5.2 (4)
C7—C8—C9—C4	2.3 (4)	C26—C27—C28—C23	−2.7 (4)
O1—C8—C9—C4	−176.6 (2)	O2—C27—C28—C23	176.6 (2)
C8—O1—C10—C11	−0.1 (4)	C27—O2—C29—C30	3.4 (4)
C8—O1—C10—N2	178.2 (2)	C27—O2—C29—N5	−174.5 (2)
O1—C10—C11—C13	179.8 (3)	N5—C29—C30—C32	−2.3 (5)
N2—C10—C11—C13	1.9 (5)	O2—C29—C30—C32	−179.7 (3)
O1—C10—C11—C12	−6.0 (5)	N5—C29—C30—C31	178.9 (3)
N2—C10—C11—C12	176.0 (3)	O2—C29—C30—C31	1.4 (5)
C8—C7—C12—C11	−3.7 (4)	C27—C26—C31—C33	126.5 (3)
C6—C7—C12—C11	178.1 (3)	C25—C26—C31—C33	−53.9 (3)
C8—C7—C12—C14	−130.8 (3)	C27—C26—C31—C30	2.3 (4)
C6—C7—C12—C14	51.0 (4)	C25—C26—C31—C30	−178.1 (2)
C10—C11—C12—C7	7.4 (4)	C29—C30—C31—C26	−4.0 (4)
C13—C11—C12—C7	−178.5 (3)	C32—C30—C31—C26	177.1 (3)
C10—C11—C12—C14	134.9 (3)	C29—C30—C31—C33	−130.6 (3)
C13—C11—C12—C14	−51.0 (4)	C32—C30—C31—C33	50.5 (4)
C7—C12—C14—C15	−132.9 (3)	C26—C31—C33—C34	149.0 (3)
C11—C12—C14—C15	101.7 (3)	C30—C31—C33—C34	−87.4 (3)
C7—C12—C14—C19	48.7 (4)	C26—C31—C33—C38	−36.7 (4)
C11—C12—C14—C19	−76.7 (4)	C30—C31—C33—C38	87.0 (3)
C19—C14—C15—C16	−0.7 (5)	C38—C33—C34—C35	−0.1 (4)
C12—C14—C15—C16	−179.2 (3)	C31—C33—C34—C35	174.5 (3)
C14—C15—C16—C17	−0.3 (5)	C33—C34—C35—C36	0.4 (5)
C15—C16—C17—C18	1.1 (5)	C34—C35—C36—C37	−0.1 (5)
C15—C16—C17—Cl1	179.8 (3)	C34—C35—C36—Cl2	179.2 (2)
C16—C17—C18—C19	−0.9 (5)	C35—C36—C37—C38	−0.6 (5)
Cl1—C17—C18—C19	−179.5 (2)	Cl2—C36—C37—C38	−179.9 (2)
C15—C14—C19—C18	1.0 (5)	C34—C33—C38—C37	−0.6 (4)
C12—C14—C19—C18	179.5 (3)	C31—C33—C38—C37	−175.1 (2)
C17—C18—C19—C14	−0.3 (5)	C36—C37—C38—C33	0.9 (5)

### Hydrogen-bond geometry (Å, º)

Cg1 and Cg2 are the centroids of the N4,C20–C23,C28 and C33–C38 rings, respectively.

**Table:**

*D*—H···*A*	*D*—H	H···*A*	*D*···*A*	*D*—H···*A*
N2—H1···N4^i^	0.84 (3)	2.34 (4)	3.172 (4)	174 (3)
N2—H2···N5^i^	0.89 (3)	2.61 (3)	3.308 (5)	136 (2)
N5—H3···N1	0.87 (3)	2.15 (3)	3.014 (3)	173 (3)
C18—H18···*Cg*1^ii^	0.93	2.80	3.650 (3)	152
C24—H24···*Cg*2^iii^	0.93	2.74	3.668 (3)	174

Symmetry codes: (i) *x*−1, *y*, *z*; (ii) −*x*−3/2, *y*−1/2, −*z*−1/2; (iii) −*x*+2, −*y*+1, −*z*+1.
